# Systematic Review of Signs and Symptoms Associated with Hematopoietic Stem Cell Transplantation-Associated Thrombotic Microangiopathy

**DOI:** 10.1016/j.jtct.2022.12.023

**Published:** 2022-12-31

**Authors:** Christopher E. Dandoy, Wan H. Tsong, Kaushik Sarikonda, November McGarvey, Miguel-Angel Perales

**Affiliations:** 1 Division of Bone Marrow Transplantation and Immune Deficiency, Cancer and Blood Disease Institute, Cincinnati Children’s Hospital Medical Center, Cincinnati, Ohio; 2 Department of Pediatrics, University of Cincinnati College of Medicine, Cincinnati, Ohio; 3 Omeros Corporation, Health Economics and Outcomes Research, Medical Affairs, Seattle, Washington; 4 BluePath Solutions, Strategic Health Economics and Outcomes Research, Los Angeles, California; 5 Department of Medicine, Adult Bone Marrow Transplantation Service, Memorial Sloan Kettering Cancer Center, New York, New York; 6 Department of Medicine, Weill Cornell Medical College, New York, New York

**Keywords:** Symptoms, Hematopoietic stem cell, transplantation, Thrombotic microangiopathy

## Abstract

Hematopoietic stem cell transplantation-associated thrombotic microangiopathy (HSCT-TMA) is a serious complication of the transplantation process that has been consistently associated with substantially greater morbidity and mortality compared with HSCT recipients who do not develop TMA. This study aimed to systematically review published signs and symptoms of HSCT-TMA and compare patients with HSCT-TMA and HSCT recipients who do not develop TMA. Publications were identified using multiple search term variations for stem cell transplantation that were entered into the PubMed, Embase, and CINAHL databases. Two reviewers screened references at the abstract level before reviewing full texts against inclusion and exclusion criteria using a PICOS-T framework. Complication proportions were grouped by organ class and then by complication type. Meta-analyses were conducted using a random-effects model in RevMan 5.4. After 2338 references were screened, a total of 30 studies were included in our analyses. The majority of studies (n = 23; 14 adult, 5 pediatric, 4 both) examined allogeneic transplantations only. Four studies examined autologous transplantation only (all pediatric), and 3 studies included both transplantation types (all pediatric). HSCT-TMA was associated with renal dysfunction (odds ratio [OR], 11.04 for adult, allogeneic and 7.35 for pediatric, all transplantations), renal failure (OR, 2.41 for adult and pediatric, allogeneic), renal replacement therapy (OR, 6.99 for pediatric, all transplantations and 60.85 for adult, allogeneic), and hypertension (OR, 5.44 for adult, allogeneic). HSCT-TMA was associated with respiratory failure (OR, 8.00 for adult and pediatric, allogeneic), pulmonary hypertension (OR, 9.86 for adult and pediatric, allogeneic), need for pleurocentesis (OR, 5.45 for pediatric, all transplantations), noninvasive ventilation (OR, 6.15 for pediatric, all transplantations), and invasive mechanical ventilation (OR, 5.18 for pediatric, all transplantations). Additionally, HSCT-TMA was associated with neurologic symptoms (OR, 2.28 for adult and pediatric, allogeneic), pericardial effusion (OR, 2.56 for adult and pediatric, allogeneic and 8.76 for pediatric, all transplantations), liver injury (OR, 3.87 for adult, allogeneic), infection (OR, 9.25 for adult, allogeneic; 2.06 for pediatric, all transplantations), gastrointestinal (GI) bleeding (OR, 7.78 for adult and pediatric, allogeneic), and acute graft-versus-host disease grade III-IV (OR, 3.29 for adult and pediatric, allogeneic). This study represents the first systematic review of HSCT-TMA signs and symptoms. Current diagnostic criteria systems involve laboratory markers for multiorgan dysfunction, including renal dys-function, liver injury, and general tissue damage. Diagnostic criteria include neurologic symptoms, increased need for transfusions, and hypertension. This study identified additional associations with HSCT-TMA, including increased pulmonary hypertension, respiratory failure, fever, GI bleeding, and pericardial effusion. These symptoms might be included for evaluation in future diagnostic criteria and current practice.

## INTRODUCTION

Hematopoietic stem cell transplantation (HSCT)-associated thrombotic microangiopathy (HSCT-TMA, or transplant-associated TMA [TA-TMA]) is a serious complication of the transplantation process that has consistently been associated with a substantial increase in morbidity and mortality compared with HSCT recipients without TMA [1–3]. An incremental mortality excess of 18% (21% versus 3%) at 1 year and 34% (40% versus 6%) at 2 years has been associated with HSCT-TMA compared with HSCT recipients who do not develop TMA [1].

HSCT-TMA is difficult to diagnose, however. One of the challenges of diagnosing HSCT-TMA is the lack of universally accepted diagnostic criteria. This has implications for both clinical management and research in the field. Although there is some overlap in diagnostic criteria for HSCT-TMA—including thrombocytopenia, presence of schistocytes, decreased hemoglobin, increased need for transfusions, increased lactate dehydrogenase (LDH), increased creatinine level or proteinuria, hypertension, normal coagulation as well as negative Coombs test, and absence of the *ADAMTS13* mutation [4–8]—individual criteria can be difficult to attribute to HSCT-TMA.

Thrombocytopenia, low hemoglobin, and the need for transfusions are difficult to attribute to HSCT-TMA during certain peritransplantation events, such as chemotherapy-induced cytopenia early after the preparatory regimen, delayed or poor engraftment, and relapse. Schistocyte readings are often inconsistent. LDH, haptoglobin, and creatinine criteria are indicators of tissue damage. Hypertension is more easily distinguished in children than in adults, who often have preexisting hypertension. Hypertension in HSCT-TMA usually occurs due to renal endothelial injury, which is particularly sensitive to HSCT-TMA. The coagulation test, Coombs test, and assessment of the *ADAMTS13* mutation are meant to exclude other disorders. Finally, some diagnostic criteria have included unexplained renal injury [5,6,8] and unexplained neurologic dys-function [5] as potential signs of HSCT-TMA.

Given the difficulties in diagnosing HSCT-TMA, the present study was conducted to systematically review publications regarding the signs and symptoms associated with HSCT-TMA to identify potential additions to future diagnostic criteria. For signs and symptoms already included in diagnostic criteria, this study aimed to quantify the proportion of HSCT-TMA patients who exhibit those criteria.

## METHODS

### Search Strategy

A systematic review of studies containing original research on HSCT-TMA-related signs and symptoms was conducted following Cochrane [9] and National Institute for Health and Care Excellence [10] guidelines on May 11, 2021. Multiple search term variations for HSCT were entered into the PubMed, Embase, and CINAHL databases.

### Study Selection

References were reviewed in a 2-level screening process in which 2 separate researchers assessed abstracts against inclusion/exclusion criteria, then reviewed full-text articles for inclusion in the analyses. Reference lists of included studies were further examined for additional publications of relevance. A third researcher adjudicated any conflicts.

Inclusion/exclusion criteria were established in a protocol and structured according to population, intervention, comparators, outcomes, study design, and time horizon (PICOS-T) (Supplementary Table S1). Included studies reported a percentage of at least 1 potential sign or symptom of TMA in HSCT recipients and were published in English between January 1, 2000, and May 11, 2021. No further restrictions were applied to the population, outcomes, or time frame, and no restrictions were applied regarding interventions and comparators.

### Data Extraction

Data were extracted according to an established Microsoft Excel data collection form by 2 trained data researchers/screeners, with quality control performed by a third lead reviewer/screener. Collected variables included the first author’s name, publication year, sample size, complication rate, description of groups compared, types of complications, any risk estimates (eg, hazard ratio, risk ratio), *P* values, and adjustment factors (ie, covariates). When available, values for HSCT recipients without TMA were extracted and hereinafter are referred to as HSCT control values.

### Data Synthesis and Analysis

Symptom proportions were grouped by organ class, then by symptom type. For studies reporting both HSCT-TMA and HSCT control values, odds ratios (ORs) and *P* values were calculated using RevMan 5.4 [11]. Meta-analyses were conducted using a random-effects model in RevMan 5.4 [12]. For studies reporting HSCT-TMA data without an HSCT control, the ranges of values are described qualitatively.

## RESULTS

### Search Results

**Figure 1.**
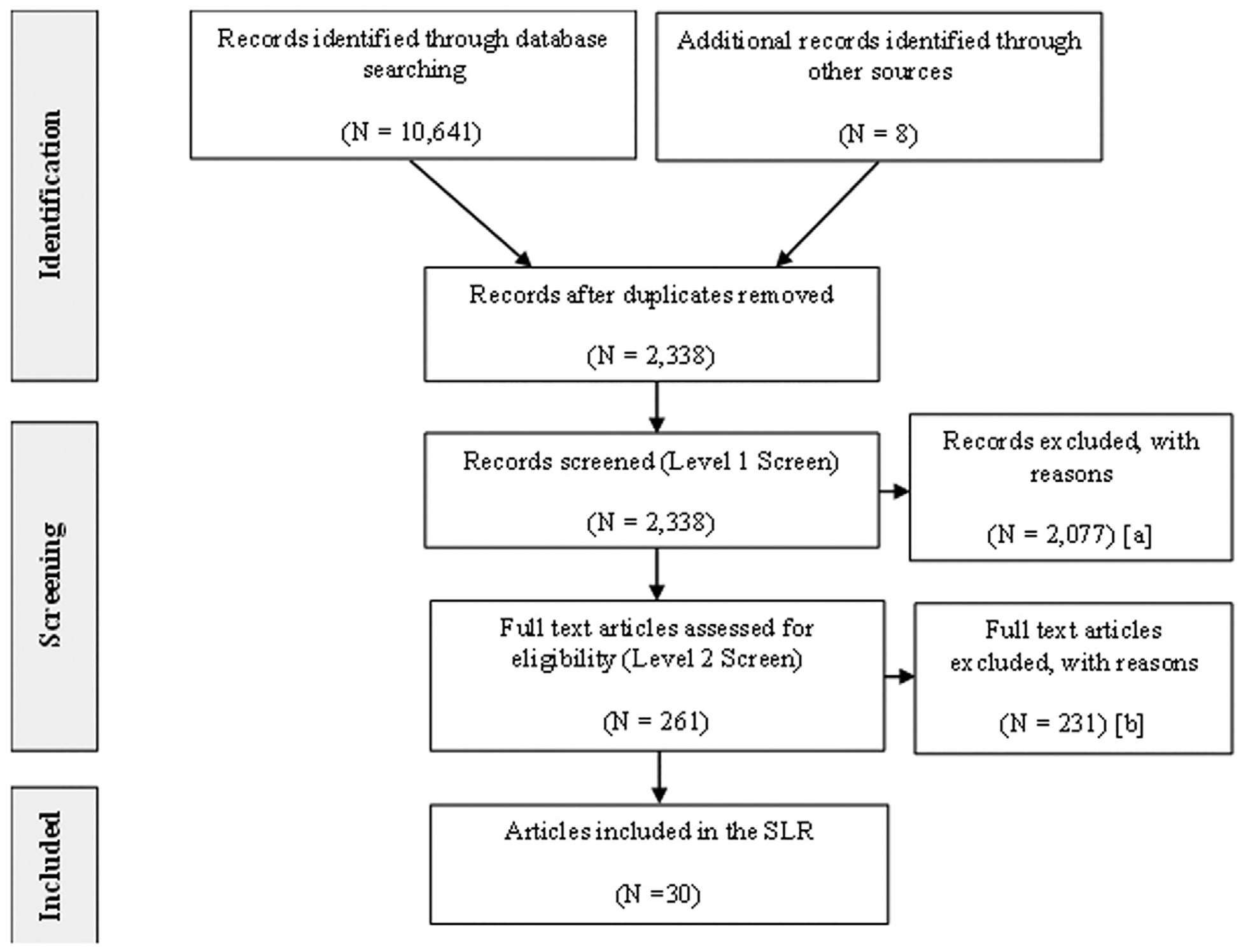
PRISMA flow chart of search results. (A) Reasons for exclusion: population, n = 481; outcomes, n = 266; study type, n = 1141; time horizon, n = 77; duplicate, n = 105; not the most recent publication, n = 7. (B) Reasons for exclusion: population, n = 20; outcomes, n = 168; study type, n = 30; time horizon, n = 1; not the most recent publication, n = 12.

The search terms produced a combined 10,641 citations across the 3 databases (Figure 1). An additional 8 citations were identified by searching through references. Following the removal of duplicates, a total of 2338 unique citations remained for screening (Figure 1). Title and abstract screening resulted in the exclusion of 2077 citations, leaving 261 for full-text screening (Figure 1). Of these 261 citations, full-text screening resulted in the exclusion of 231 citations and the inclusion of 30 citations (Figure 1).

### Study Characteristics

The characteristics of the 30 included studies are shown in Table 1. Most of the publications (n = 22 [10 adult, 10 pediatric, 2 both]) covered patient populations treated in the United States. The remaining 8 publications included patients treated in France (n = 1, combined adult/pediatric), Switzerland (n = 2, adult and combined adult/pediatric), Greece (n = 1, pediatric), Turkey (n = 1, pediatric), Israel (n = 1, adult), China (n = 1, adult), and Japan (n = 1, adult). Patient enrollment periods across publications spanned 1994 to 2019. Most of the studies (n = 23 [14 adult, 5 pediatric, 4 both]) examined allogeneic transplantation only. Four studies examined autologous transplantations only (all pediatric), and 3 studies examined combined allogeneic and autologous transplantations (all pediatric). Fifteen publications did not mention any restrictions in their enrollment criteria; the other 15 publications evaluated subpopulations of the total HSCT-TMA population, including 4 that allowed treatment with eculizumab, 3 that selected according to the specific type of graft-versus-host disease (GVHD) prophylaxis used, 2 that reported neuroblastoma, 2 that detailed intestinal symptoms, and 1 each that reported engraftment status, treatment with plasma exchange, monitoring for von Willebrand factor levels, and T cell-depleted HSCT.

### Study Findings

**Figure 2.**
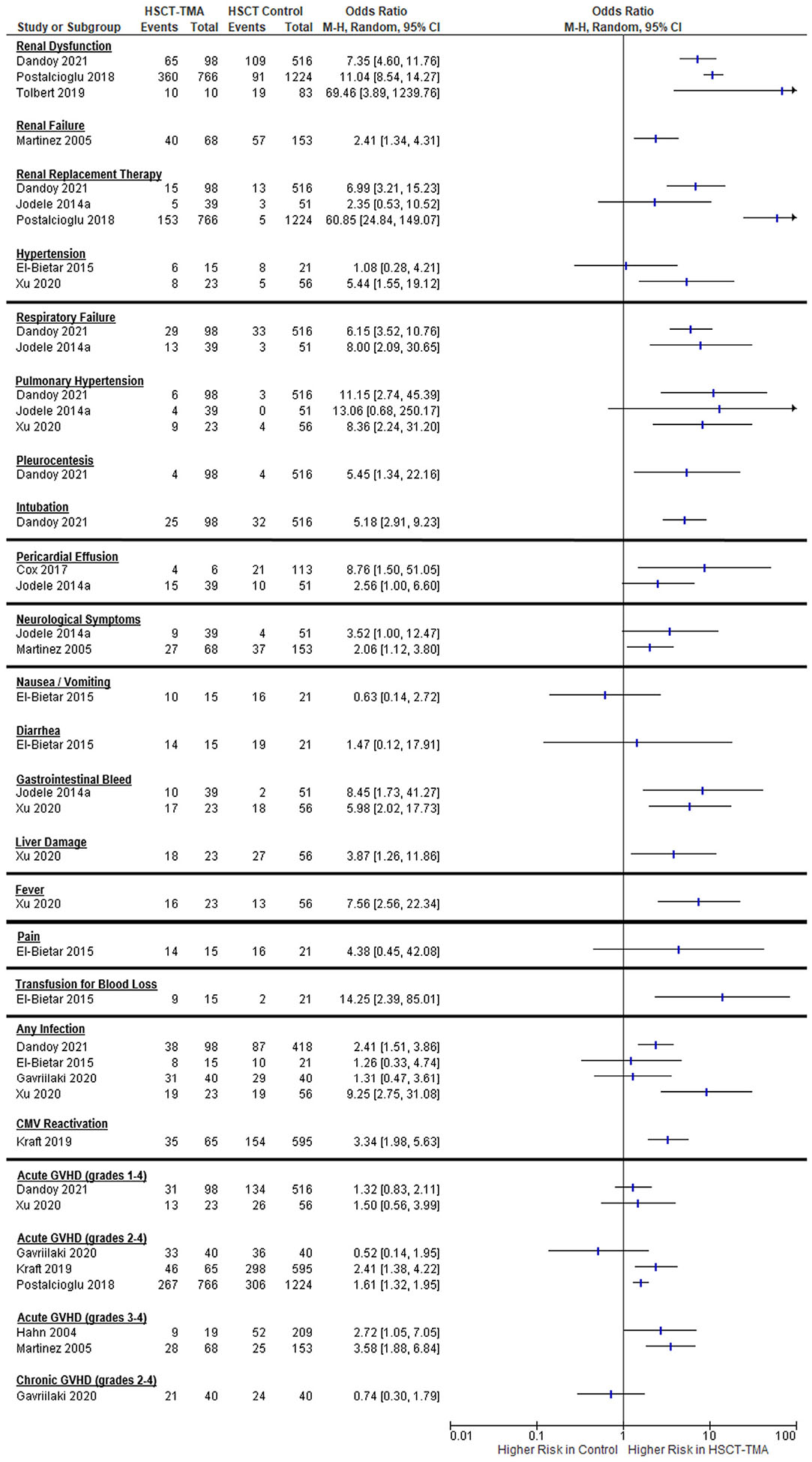
Associations of HSCT-TMA with specific signs and symptoms.

Associations between HSCT-TMA and specific signs and symptoms are presented in Figure 2 and Table 2. Arithmetic risk differences are presented in Table 3.

#### Renal symptoms

Renal symptoms are presented in Supplementary Table S2. Renal dysfunction, abnormality, or acute kidney injury was reported across 16 studies, with values ranging from 10% to 100%. Three studies reported HSCT control values for renal dysfunction, with arithmetic increases ranging from 40% to 45% in the 2 studies with no population restrictions (OR, 7.35; 95% confidence interval [CI], 4.60 to 11.76 and OR, 11.04; 95% CI, 8.54 to 14.27) to 77% in the study restricted to pediatric neuroblastoma patients (OR, 69.46; 95% CI, 3.89 to 1239.76). In a study of pediatric and adult HSCT recipients, renal failure was reported in 59% of patients with HSCT-TMA and in 37% of HSCT recipients without TMA (OR, 2.41; 95% CI, 1.34 to 4.31) [13]. Renal replacement therapy (RRT) was reported in 13 studies, with percentages ranging from 11% to 53% in HSCT-TMA patients. Three studies (2 primarily pediatric, 1 adult) reported RRT percentages ranging from .4% to 6% for HSCT controls and values 7% to 20% higher in HSCT-TMA patients. The association of HSCT-TMA with the need for RRT was statistically significant in the 2 larger studies (OR, 60.85 and 6.99) but not in the smaller pediatric study (n = 39 TMA; n = 51 non-TMA) (Figure 2). Considerable heterogeneity among these studies was observed (*I*^2^ = 91%), caused mostly by the difference between the adult and pediatric data (*I*^2^ = 39% without adult data). Edema was reported in 27% of HSCT-TMA patients. Hypertension was reported in 7 studies, with percentages in HSCT-TMA patients ranging from 32% to 100%. Two studies reported HSCT control data, with percentages ranging from 9% to 38% for hypertension, but the results were inconsistent with substantial heterogeneity observed (*I*^2^ = 67%) [14,15]. Chronic kidney disease was reported in 14% to 93% of HSCT-TMA patients, end-stage renal disease was reported in 9% of adult HSCT-TMA patients, and kidney transplantation was performed in 33% of pediatric HSCT-TMA patients, all of whom were treated with plasma exchange (Supplementary Table S2).

#### Pulmonary symptoms

Pulmonary symptoms are presented in Supplementary Table S3. In a study of 10 pediatric patients, poor pulmonary function was reported in 40% of HSCT-TMA patients. Across 6 studies, pulmonary hypertension was reported in 6% to 39% of HSCT-TMA patients. In the 3 studies with HSCT controls, the percent of pulmonary hypertension ranged from 0 to 7% in the controls, compared with 6% to 39% in HSCT-TMA patients. Our meta-analysis indicated that HSCT-TMA patients had an almost 10-fold greater odds of having pulmonary hypertension compared with HSCT controls (OR, 9.86; 95% CI, 3.95 to 24.57). The largest arithmetic difference occurred in the adult population (39% versus 7%). Pulmonary hemorrhage was reported in 5% to 17% of HSCT-TMA patients; however, no data were available for HSCT controls in those studies. Across 3 studies that did not specify the type of treatment for respiratory failure, 15% to 44% of HSCT-TMA patients had respiratory failure. In the study that reported HSCT control data, HSCT-TMA patients had an 8-fold increase in respiratory failure (33% versus 6%; OR, 8.00; 95% CI, 2.09 to 30.65). In 2 pediatric studies, pleurocentesis was reported in 3% to 4% of HSCT-TMA patients. One study provided a percentage of pleurocentesis for HSCT controls (.8%), indicating that HSCT-TMA (4%) was associated with a 5-fold increase in the risk of pleurocentesis (OR, 5.45; 95% CI, 1.34 to 22.16). Noninvasive ventilation was reported in 11% of adult allogeneic HSCT-TMA patients [16] and in 30% of pediatric HSCT-TMA patients, compared with 6% of the HSCT controls (OR, 6.15; 95% CI, 3.52 to 10.76) [17]. Across 4 studies (2 adult and 2 pediatric), invasive mechanical ventilation was reported in 29% to 44% of HSCT-TMA patients. One of the pediatric studies reported HSCT control data, indicating that HSCT-TMA was associated with a 5-fold increase in intubation (26% versus 6%; OR, 5.18; 95% CI, 2.91 to 9.23).

#### Cardiovascular symptoms

Cardiovascular symptoms of HSCT-TMA are presented in Supplementary Table S4. Poor cardiac function was reported in 20% of HSCT-TMA patients, and pericarditis was reported in 17% of HSCT-TMA patients. Pericardial effusion was reported in 6 studies, with values ranging from 9% to 92%. In studies with HSCT controls, pericardial effusion was present in .6% to 20% of controls and in 9% to 67% in HSCT-TMA patients. Moderate heterogeneity was observed between these studies (*I*^2^ = 31%). The association between HSCT-TMA and pericardial effusion was much larger in the pediatric study that included autologous and allogeneic transplantation recipients (OR, 8.76; 95% CI, 1.50 to 51.05) compared with the combined adult and pediatric study, which included only allogeneic transplantation recipients (OR, 2.56; 95% CI, 1.00 to 6.60). Cardiac tamponade was reported in 17% to 22% of HSCT-TMA patients.

#### Neurologic symptoms

Central nervous system symptoms are presented in Supplementary Table S5. Encephalopathy was reported in 9% to 33% of HSCT-TMA patients, and seizures were reported in 11% to 19%. Any neurologic symptoms were reported collectively in 15 studies, with values in HSCT-TMA patients ranging from 0 to 74%. Control values were reported in 2 pediatric studies, both of which demonstrated an approximately 15% to 16% higher percentage in the HSCT-TMA patients compared with HSCT controls (23% versus 8% [6] and 40% versus 24% [13]). The odds of having neurologic symptoms were 2-fold higher in the HSCT-TMA patients (OR, 2.28; 95% CI, 1.32 to 3.96). Both the pediatric and adult studies had similar arithmetic risk differences (15% versus 16%), but the adult study had a higher percentage in the controls (24% versus 8%). CNS bleeding was reported in 2% to 15% of HSCT-TMA pediatric patients, and stroke was reported in 11% of HSCT-TMA adult patients.

#### Gastrointestinal and hepatic symptoms

Gastrointestinal (GI) and hepatic symptoms of HSCT-TMA are reported in Supplementary Table S6. Although nausea or vomiting, (67%), diarrhea (93%), and abdominal pain (93% versus 76% in controls) were reported in HSCT-TMA patients, no association with HSCT-TMA was identified when compared to the percentage in HSCT controls (OR, .63 [95% CI, .14 to 2.72], 1.47 [95% CI, .12 to 17.91], and 4.38 [95% CI, .45 to 42.08], respectively). In 5 studies, GI bleeding was reported in 8% to 74% of HSCT-TMA patients. HSCT control data in 3 of these studies indicated that HSCT-TMA was associated with an almost 8-fold greater risk of GI bleeding (OR, 7.78; 95% CI, 3.49 to 17.33). Liver injury was reported in 10% to 78% of HSCT-TMA patients. HSCT control data were available in 1 of the studies, and HSCT-TMA was associated with an almost 4-fold increase in liver damage (OR, 3.87; 95% CI, 1.26 to 11.86).

#### Other symptoms

Other symptoms of HSCT-TMA are reported in Supplementary Table S7. Multiorgan dysfunction was reported in 70% to 84% of HSCT-TMA patients in 2 studies. In 1 study of severe HSCT-TMA patients, retinitis was reported in 8% of these patients.

#### Infection

Reported infection values are listed in Supplementary Table S8. Across the publications, infections were reported in 39% to 83% of HSCT-TMA patients. In 4 studies with control data, the underlying percentage of infection ranged from 21% to 73% in HSCT controls and from 39% to 83% in HSCT-TMA patients. Substantial heterogeneity (*I*^2^ = 58%) was observed between the adult study (OR, 9.25; 95% CI, 2.75 to 31.08) and the pediatric studies (OR, 2.06; 95% CI, 1.37 to 3.09), with a much greater association between HSCT-TMA and any infection in the adult study. Regarding specific infections in HSCT-TMA patients, cytomegalovirus (CMV) infection was reported in 11% to 54% of patients, *Clostridium difficile* infection in 11%, *Enterococcus* infection in 22%, *Staphylococcus* infection in 22%, and meningitis in 2%. In a study in which HSCT control data were reported, HSCT-TMA was associated with a 3-fold increase in CMV reactivation [54% vs. 26%; OR 3.34; 95% CI, 1.98–5.63]. Fever was reported in 70% of adult HSCT-TMA patients versus 23% in control HSCT recipients, resulting in a >7-fold increase in fever for HSCT-TMA patients (OR, 7.56; 95% CI, 2.56 to 22.34).

#### GVHD

Reported GVHD values are listed in Supplementary Table S9. Two studies reported the proportion of HSCT-TMA patients with acute GVHD onset after TMA (10% among adult patients with GVHD and HSCT-TMA, 23% in pediatric patients). Most studies reported the proportion of acute GVHD that occurred concurrently with HSCT-TMA, with values ranging from 32% to 100%. Seven studies reported acute GVHD values for HSCT controls, with differences ranging from −8% to 6% in the 2 pediatric studies to increases of 10% to 21% for HSCT-TMA in the adult studies. Studies reporting all grades of acute GVHD showed no significant increase in acute GVHD associated with HSCT-TMA (OR, 1.35; 95% CI, .89 to 2.06). Studies reporting acute GVHD grade II-IV demonstrated substantial heterogeneity (*I*^2^ = 58%), with the adult study showing an association (OR, 1.80; 95% CI, 1.26 to 2.59) and the pediatric study showing no association (OR, .52; 95% CI, .14 to 1.95). Two studies reporting combined adult and pediatric data showed an association for acute GVHD grade III-IV (OR, 3.29; 95% CI, 1.92 to 5.61). The percentage of acute GVHD was 16% to 25% in HSCT controls and 41% to 47% in HSCT-TMA patients. Severe bleeding related to GI GVHD was reported in 8% of HSCT-TMA patients. Chronic GVHD was reported in 50% to 88% of HSCT-TMA patients; however, the 1 study that reported a comparison with HSCT controls showed no association with HSCT-TMA (OR, .74; 95% CI, .30 to 1.79).

## DISCUSSION

This study represents the first systematic review of the signs and symptoms associated with HSCT-TMA across multiple organ systems. The results support the concept that HSCT-TMA manifests as a constellation of symptoms, including increases in renal dysfunction and the need for RRT, risk of respiratory failure and the need for intubation and mechanical ventilation, risk of pulmonary hypertension, risk of neurologic symptoms, risk of grade III-IV acute GVHD, risk of infection in untreated patients, risk of pericardial effusion, risk of GI bleeding and transfusion for blood loss, and risk of liver damage. In adult patients, evidence was available exclusively in allogeneic transplantation recipients, and associations were strongest between HSCT-TMA and RRT (OR, 60.85), renal dysfunction (OR, 11.04), pulmonary hypertension (OR, 9.86) and any infection (OR, 9.25). Arithmetic risk differences were greatest for any infection (49%), fever (46%), GI bleeding (42%), and renal dysfunction (40%). In pediatric patients, most of the available evidence was from allogeneic or combined populations of allogeneic and autologous HSCT recipients, and associations were strongest between HSCT-TMA and renal dysfunction (OR, 69.46 for autologous, 7.35 for combined), pulmonary hypertension (OR, 9.86), and pericardial effusion (OR, 8.76). Arithmetic risk differences were greatest for renal dysfunction (77% autologous, 45% combined), GI bleeding (50%), and pulmonary hypertension (32%).

Current diagnostic criteria involve laboratory markers for renal dysfunction, liver injury, and general tissue damage. Some diagnostic criteria include neurologic symptoms, increased need for transfusions, and hypertension. Pulmonary hypertension, respiratory failure, fever, GI bleeding, and pericardial effusion are not explicitly mentioned in diagnostic criteria because they are primarily associated with organ injury. These symptoms might be included for evaluation in future diagnostic criteria and current practice.

The strengths of this study lie in its systematic search for published data and the meta-analyses, which used a standard method to analyze data across studies as opposed to relying on varying statistical methods from separate studies. A limitation of this study is that further research at the patient level is needed to map the number of symptoms present to the probability of having HSCT-TMA, likely further improving diagnostic accuracy. Patient-level analysis could aid causal analysis of end-organ injury from HSCT-TMA. Overall, this study provides important additional information on when HSCT-TMA should be suspected and when further investigation is warranted, which may lead to improved diagnostic accuracy and, ultimately, better patient outcomes.

## Supplementary Material

1

## Figures and Tables

**Table 1:** Study Characteristics of the Included Publications

First Author, Year	Center	Patient Year.Month	HSCT, N	TMA,n	No TMA, n	Special Population	Adult/ Pediatric	HSCT Type
Cox, 2017 [18]	Lucile Packard Children’s Hospital, CA	2010.01–2013.12	119	6	113	No restrictions	Pediatric	Both
Cutler, 2005 [19]	Dana-Farber Cancer Institute, MA	1997–2003	327	21	306	Cyclosporine or tacrolimus as immuno-suppressive regimen; exclusions for prior HSCT, T cell depletion, baseline creatinine >1.5 mg/dL, or veno-occlusive disease of liver	Adult	Allogeneic
Dandoy, 2021 [17]	13 HSCT centers in the United States	2016–2019	614	98	516	No restrictions	Pediatric	Both
De Fontbrune, 2015 [19]	9 HSCT centers in France	2010.03–2013.12	NR	12	NR	All ECU; severe HSCT-TMA (neurologic and/or renal involvement)	Both	Allogeneic
El-Bietar, 2015 [15]	Cincinnati Children’s Hospital, OH	2008.06–2012.12	50	15	35	Intestinal HSCT-TMA	Pediatric	Allogeneic
Erbey, 2010 [21]	Cukurova and Hacettepe Universities, Turkey	2006.01–2008.06	50	3	47	No restrictions	Pediatric	Allogeneic
Gavriilaki, 2020 [22]	G. Papanicolaou Hospital, Greece	2014–2017	80	40	40	No restrictions (TMA to non-TMA ratio controlled by case-control design)	Pediatric	Allogeneic
Glezerman, 2010 [23]	Memorial Sloan Kettering, NY	2001.01–2006.12	100	11	89	T cell-depleted HSCT: cohort A without TBI, cohort B with TBI	Adult	Allogeneic
Hahn, 2004 [24]	Roswell Park Cancer Institute, NY	1994.04–1997.10; 1997.11–2000.11	228	19	209	No restrictions	Both	Allogeneic
Heybeli, 2020 [25]	Mayo Clinic, MN	2000.01–2019.06	1451	84	1367	No restrictions	Adult	Allogeneic
Imus, 2020 [16]	Johns Hopkins University, MD	2015.01–2018.08	678	9	669	All received high-dose post-HSCT cyclophosphamide for GVHD prophylaxis	Adult	Allogeneic
Jodele, 2014a [6]	Cincinnati Children’s Hospital, OH	2010.09–2011.12	90	39	51	No restrictions	Both	Allogeneic
Jodele, 2014b [26]	Cincinnati Children’s Hospital, OH	2012.01–2013.05	NR	6	NR	All ECU	Pediatric	Autologous
Jodele, 2018 [27]	Cincinnati Children’s Hospital, OH	2005.01–2015.12	60	13	47	High-risk neuroblastoma	Pediatric	Autologous
Jodele, 2020 [28]	Cincinnati Children’s Hospital, OH; Children’s Hospital of Philadelphia, PA; Children’s Hospital Los Angeles, CA	2012.03–2018.10	566	64	502	All ECU	Pediatric	Both
Kraft, 2019 [3]	University of Basel, Switzerland	2006.12–2016.04	660	65	595	No restrictions	Adult	Allogeneic
Li, 2019 [29]	Seattle Children’s Hospital Fred Hutch, WA	2006.01– 2015.12	2145	192	1953	No restrictions	Adult	Allogeneic
Martinez, 2005 [13]	Basel University Hospitals, Switzerland	1995–2002	221	68	153	No restrictions	Both	Allogeneic
Nakamae, 2006 [30]	Osaka City University, Japan	1994.04–2004.12	123	22	101	No restrictions	Adult	Allogeneic
Oran, 2007 [31]	MD Anderson, TX	1998.01–2004.12	1219	66	1153	All engrafted with non-T cell- depleted transplantation and received tacrolimus-based GVHD prophylaxis	Adult	Allogeneic
Postalcioglu, 2018 [32]	Dana Farber Cancer Institute, MA	2005.01–2013.12	1990	766	1224	No restrictions	Adult	Allogeneic
Rosenthal, 2011 [33]	City of Hope, CA	2004.01–2008.04	41	10	31	All treated with sirolimus and tacrolimus	Pediatric	Allogeneic
Rudoni, 2018 [34]	MD Anderson, TX	2013.01–2016.09	NR	10	NR	All ECU	Adult	Allogeneic
Sarkodee-Adoo, 2003 [35]	University of Maryland Greenebaum Cancer Center, MD	1995.07–1999.06	55	11	44	No restrictions	Adult	Allogeneic
Sartain, 2019 [36]	Baylor Texas Children’s Hospital, TX	2007.01– 2017.06	NR	15	NR	All plasma exchange treated	Pediatric	Allogeneic
Schoettler, 2019 [37]	Dana-Farber/Boston Children’s Cancer and Blood Disorders Center, MA	2008.01– 2018.07	243	9	234	No restrictions	Pediatric	Autologous
Shimoni, 2004 [38]	Chaim Sheba Medical Center, Israel	2000.07– 2003.12*	147	22	125	No restrictions	Adult	Allogeneic
Tolbert, 2019 [39]	University of California San Francisco Benioff Children’s Hospitals, CA	2000–2017	141	10	131	High-risk neuroblastoma	Pediatric	Autologous
Wall, 2018 [40]	Ohio State University Comprehensive Cancer Center, OH	2008.10–2016.10	124	84	40	Patients with grade III-IV acute GI GVHD and at least 1 endoscopy with biopsy	Adult	Allogeneic
Xu, 2020 [14]	Guangdong Provincial People’s Hospital, China	2016.08–2018.06	79	23	56	Patients monitored serially for von Willebrand factor levels	Adult	Allogeneic

* End date not stated in published article; estimated based on article submission date. ECU indicates eculizumab; TBI, total body irradiation.

**Table 2:** ORs (95 CIs) for Symptoms by Population

	Allogeneic HSCT		Combined HSCT	Autologous HSCT
**Adult**	**Renal dysfunction: 11.04 (8.54–14.27)** [32] **RRT: 60.85 (24.84–149.07)** [32] **Hypertension: 5.44 (1.55–19.12)** [14] **Liver injury: 3.87 (1.26–11.86)** [14] **Any infection: 9.25 (2.75–31.08)** [14] **CMV reactivation: 3.34 (1.98–5.63)** [3] **Fever: 7.56 (2.56–22.34)** [14] **Acute GVHD grade II-IV: 1.80 (1.26–2.59)** [3,32]	**Pulmonary hypertension: 9.86 (3.95–24.57)** [17,6,14] **GI bleeding: 7.78 (3.49–17.33)** [15,6,14] Acute GVHD grade I-IV: 1.35 (.89–2.06) [17,14]		
Combined	**Renal failure: 2.41 (1.34–4.31)** [13] RRT: 2.35 (.53–10.52) [6] **Respiratory failure 8.00 (2.09–30.65)** [6] **Pericardial effusion: 2.56 (1.00–6.60)** [6] **Neurologic symptoms: 2.28 (1.32–3.96)** [6, 13] **Acute GVHD grade III-IV: 3.29 (1.92–5.61)** [24, 13]			
Pediatric	Hypertension: 1.08 (.28–4.21) [15] Nausea/vomiting: .63 (.14–2.72) [15] Diarrhea: 1.47 (.12–17.91) [15] Abdominal pain: 4.38 (.45–42.08) [15] Acute GVHD grade II-IV: .52 (.14–1.95) [22] Chronic GVHD: .74 (.30–1.79) [22]		**Renal dysfunction: 7.35 (4.60–11.76)** [17] **RRT: 6.99 (3.21–15.23)** [17] **Pleurocentesis: 5.45 (1.34–22.16)** [17] **Noninvasive ventilation: 6.15 (3.52–10.76)** [17] **Invasive ventilation: 5.18 (2.91–9.23)** [17] **Pericardial effusion: 8.76 (1.50–51.05)** [18]	**Renal dysfunction: 69.46 (3.89–1239.76)** [39]
	**Any infection: 2.06 (1.37–3.09)** [17,15,22]			

Variables with statistically significant differences (*P* < .05) are shown in bold type.

**Table 3:** Arithmetic Risk Differences Associated with HSCT-TMA

	Allogeneic HSCT		Combined HSCT	Autologous HSCT
Adult	**Renal dysfunction: 47% vs 7% = 40%** [32] **RRT: 20% vs .4% = 20%** [32] **Hypertension: 35% vs 9% = 26%** [14] **Liver injury: 78% vs 48% = 30%** [14] **Any infection: 83% vs 34% = 49%** [14] **CMV reactivation: 54% vs 26% = 28%** [3] **Fever: 70% vs 23% = 46%** [14] **Acute GVHD grade II-IV: 35% vs 25% = 10%** [32]**; 71% vs 50% = 21%** [3]	**Pulmonary hypertension: P : 6% vs 1% = 6%** [17] **B: 10% vs 0% = 10%** [6] **A: 39% vs 7% = 32%** [14] **GI bleeding: P: 60% vs 10% = 50%** [15] **B: 26% vs 4% = 22%** [6] **A: 74% vs 32% = 42%** [14] Acute GVHD grade I-IV: P: 32% vs 26% = 6% [17] A: 57% vs 46% = 10% [14]		
Combined	**Renal failure: 59% vs 37% = 22%** [13] RRT: 13% vs 6% = 7% [6] **Respiratory failure 33% vs 6% = 27%** [6] **Pericardial effusion: 38% vs 20%= 19%** [6] **Neurologic symptoms: P: 23% vs 8% = 15%** [6]; **A: 40% vs 24% = 16%** [13] **Acute GVHD grade III-IV: A: 41% vs 16% = 25%** [13]; **B: 47% vs 25% = 22%** [24]			
Pediatric	Hypertension: 40% vs 38% = 2% [15] Nausea/vomiting: 67% vs 76% = −10% [15] Diarrhea: 93% vs 90% = 3% [15] Abdominal pain: 93% vs 76% = 17% [15] Acute GVHD grade II-IV: 83% vs 90% = −8% [22] Chronic GVHD: 53% vs 60% = −8% [22]		**Renal dysfunction: 66% vs 21%=45%** [17] **RRT: 15% vs 3% = 13%** [17] **Pleurocentesis: 4% vs 1% = 3%** [17] **Noninvasive ventilation: 30% vs 6% = 23%** [17] **Invasive ventilation: 26% vs 6% = 19%** [17] **Pericardial effusion: 67% vs 19% = 48%** [18]	**Renal dysfunction: 100% vs 23% = 77%** [39]
	**Any infection: P: 39% vs 21% = 18%** [17]; **P: 53% vs 48% = 6%** [15]; **P: 78% vs 73% = 5%** [22]			

Variables with statistically significant differences (*P* < .05) are shown in bold type.

A indicates adult; B, both; P, pediatric.
