# Supplementary material for: Systematic Review of Signs and Symptoms Associated with Hematopoietic Stem Cell Transplantation-Associated Thrombotic Microangiopathy
Source: Transplant Cell Ther. Author manuscript; Available in PMC 2024 Jul 29. (PMC11284894; doi:10.1016/j.jtct.2022.12.023)
Supplement: 1 [file NIHMS2011563-supplement-1.pdf]

Supplemental Tables for “Systematic Review of Signs and Symptoms Associated with Hematopoietic Stem Cell Transplantation-Associated Thrombotic Microangiopathy”

Dandoy et al. *Transplantation and Cellular Therapy*

Table S1. PICOS-T Inclusion / Exclusion Criteria

	Inclusion Criteria	Exclusion Criteria
**Population**	• Patients with HSCT-TMA (overall population)<br>• All ages<br>Among HSCT-TMA population, subpopulations of interest include:<br>▪ Patients with possible, probable, or definite HSCT-TMA<br>▪ Patients with mild, moderate, or severe HSCT-TMA<br>▪ Patients with non-persistent or persistent HSCT-TMA<br>▪ Patients with and without concomitant graft-versus-host disease (GvHD)<br>▪ Patients responsive or non-responsive to treatment (including off-label) and/or patients with resolved or unresolved HSCT-TMA	▪ Non-human
**Interventions**	Although no approved or guideline-recommended treatments are available, literature on patients receiving treatments for HSCT-TMA with the following were considered:<br>▪ Supportive care<br>▪ Narsoplimab (OMS721)<br>▪ Eculizumab<br>▪ Defibrotide	▪ None
**Comparators**	Although no approved or guideline-recommended treatments are available, literature on patients receiving treatments for HSCT-TMA with the following were considered:<br>▪ Supportive care<br>▪ Narsoplimab (OMS721)<br>▪ Eculizumab<br>▪ Defibrotide	▪ None
**Outcomes**	▪ Signs and symptoms in HSCT-TMA patients expressed as a percent of HSCT-TMA patients	
**Study type**	Includes:<br>▪ Randomized clinical trial<br>▪ Observational study<br>▪ Epidemiologic study<br>▪ Burden of illness study<br>▪ Cost of illness study<br>▪ Meta-analysis / indirect treatment comparisons	▪ Guidelines<br>▪ Expert opinion / reviews<br>▪ Consensus statement<br>▪ Comments<br>▪ Case reports<br>▪ Non-English language<br>▪ Molecular, cellular studies<br>▪ Pharmacokinetic studies<br>▪ Pharmacodynamic studies
**Time horizon of search**	▪ Studies published between 2000 to search date (May 11, 2021).	Not applicable

GvHD = graft versus host disease, HSCT-TMA = hematopoietic stem cell transplant-associated thrombotic microangiopathy

Table S2. Renal Symptoms

Author Year	Adult / Peds	HSCT Type	Special Population	Complication	TMA (N)	TMA %	non-TMA (N)	non-TMA %	Diff
**Renal Dysfunction**									
Rosenthal 2011	Peds	Allo	all SIR & TAC	Poor renal function	10	10%	31	NR	
Oran 2007	Adult	Allo	all engrafted w/ non-T-cell depleted transplant and had TAC-based GVHD prophylaxis	Renal abnormality >=50% increase BL creatinine	66	33%	1153	NR	
Li 2019	Adult	Allo	no restrictions	Acute kidney injury	192	46%	1953	NR	
Postalcioglu 2018	Adult	Allo	no restrictions	Kidney dysfunction	766	47%	1224	7%	40%
Nakamae 2006	Adult	Allo	no restrictions	Renal dysfunction	22	64%	101	NR	
Kraft 2019	Adult	Allo	no restrictions	Proteinuria (relevant)	65	66%	595	NR	
Imus 2020	Adult	Allo	all pts received high-dose post-HSCT cyclophosphamide for GVHD prophylaxis	Acute kidney injury	9	89%	669	NR	
Rudoni 2018	Adult	Allo	all ECU	Renal dysfunction	10	90%		NR	
de Fontbrune 2015	Both	Allo	all ECU; severe HSCT-TMA (neuro and/or renal involvement)	Organ injury: kidney function	12	92%	NR	NR	
Kraft 2019	Adult	Allo	no restrictions	New or worsening hypertension	65	32%	595	NR	
Jodele 2018	Peds	Auto	Neuroblastoma	Proteinuria	13	100%	NR	NR	
Tolbert 2019	Peds	Auto	Neuroblastoma	Proteinuria	10	100%	83	23%	77%
Dandoy 2021	Peds	Both	no restrictions	Acute kidney injury	98	66%	516	21%	45%
Schoettler 2019	Peds	Auto	Mostly neuroblastoma (78%)	Acute kidney injury	9	89%	NR	NR	
Hahn 2004	Both	Allo	no restrictions	Renal insufficiency	19	89%	209	NR	
Sarkodee-Adoo 2003	Adult	Allo	no restrictions	Renal insufficiency	11	100%	44	NR	
**Renal Failure**									
Martinez 2005	Both	Allo	no restrictions	Renal failure	68	59%	153	37%	22%
**Renal Replacement Therapy (RRT)**									
Li 2019	Adult	Allo	no restrictions	RRT (hemodialysis)	192	11%	1953	NR	
Jodele A2014a	Both	Allo	no restrictions	RRT (acute dialysis)	39	13%	51	6%	7%
Dandoy 2021	Peds	Both	no restrictions	RRT (including continuous RRT)	98	15.3%	516	2.5%	12.8%

Author Year	Adult / Peds	HSCT Type	Special Population	Complication	TMA (N)	TMA %	non-TMA (N)	non-TMA %	Diff
De Fontbrune 2015	Both	Allo	all ECU; severe HSCT-TMA (neuro and/or renal involvement)	RRT (hemodialysis)	12	17%	NR	NR	
Postalcioglu 2018	Adult	Allo	no restrictions	RRT	766	20%	1224	0.4%	20%
Heybeli 2020	Adult	Allo	no restrictions	RRT	84	21%	1367	NR	
Jodele 2020	Peds	Both	all ECU	RRT	64	23%	502	NR	
Jodele 2014b	Both	Allo	all ECU	RRT	6	33%		NR	
Sartain 2019	Peds	Allo	all PE treated	RRT	15	40%		NR	
Imus 2020	Adult	Allo	all pts received high-dose post-HSCT cyclophosphamide for GVHD prophylaxis	RRT	9	44%	669	NR	
Hahn 2004	Both	Allo	no restrictions	RRT	19	53%	209	NR	
Schoettler 2019	Peds	Auto	Mostly neuroblastoma (78%)	RRT (acute renal failure)	9	22%	NR	NR	
Jodele 2018	Peds	Auto	Neuroblastoma	RRT	13	23%	NR	NR	
**Edema**									
Glezerman 2010	Adult	Allo	T-cell depleted HSCT	Edema	11	27%	89	NR	
**Hypertension**									
Xu 2020	Adult	Allo	monitored serially for von Willebrand Factor levels	Hypertension	23	35%	56	9%	26%
El-Bietar 2015	Peds	Allo	intestinal GVHD	Hypertension	15	40%	21	38%	2%
Schoettler 2019	Peds	Auto	Mostly neuroblastoma (78%)	Hypertension	9	56%	NR	NR	
Jodele 2018	Peds	Auto	Neuroblastoma	Hypertension	13	100%	NR	NR	
Glezerman 2010	Adult	Allo	T-cell depleted HSCT	New or worsening hypertension	11	64%	89	NR	
Kraft 2019	Adult	Allo	No restrictions	New or worsening hypertension	65	32%	595	NR	
Jodele 2014b	Peds	Auto	all ECU	Systemic hypertension	6	100%	NR	NR	
**Chronic Kidney Disease**									
Cutler 2005	Adult	Allo	Cyclosporin or tacrolimus as immunosuppressive regimen	Chronic kidney disease	21	14%	306	NR	
Sartain 2019	Peds	Allo	all PE treated	Severe chronic kidney disease	15	33%	NR	NR	
Sartain 2019	Peds	Allo	all PE treated	Chronic kidney disease	15	93%	NR	NR	
**End Stage Renal Disease and Kidney Transplant**									

Author Year	Adult / Peds	HSCT Type	Special Population	Complication	TMA (N)	TMA %	non-TMA (N)	non-TMA %	Diff
Glezerman 2010	Adult	Allo	T-cell depleted HSCT	End stage renal disease	11	9%	89	NR	
Sartain 2019	Peds	Allo	all PE treated	Kidney transplant	15	33%	NR	NR	

Allo = allogeneic, Auto = autologous, ECU = eculizumab, DNA = deoxyribonucleic acid, GVHD = graft versus host disease, HSCT = hematopoietic stem cell transplant, NR = not reported, PE = plasma exchange, Peds = pediatric, pts = patients, SIR = sirolimus, TAC = tacrolimus, TMA = thrombotic microangiopathy

Table S3. Pulmonary Symptoms

Author Year	Adult / Peds	HSCT Type	Special Population	Complication	TMA (N)	TMA %	non-TMA (N)	non-TMA %	Diff
**Poor Pulmonary Function**									
Rosenthal 2011	Peds	Allo	all SIR & TAC	Poor pulmonary function	10	40%	31	NR	
**Pulmonary Hypertension**									
Dandoy 2021	Peds	Both	no restrictions	Pulmonary hypertension	98	6%	516	1%	6%
Jodele 2014a	Both	Allo	no restrictions	Pulmonary hypertension	39	10%	51	0%	10%
Jodele 2020	Peds	Both	all ECU	Pulmonary hypertension	64	17%	502	NR	
Jodele 2018	Peds	Auto	Neuroblastoma	Pulmonary hypertension requiring therapy	13	23%	NR	NR	
Jodele 2020	Peds	Both	all ECU	Pulmonary hypertension needing inhaled NO	64	17%	502	NR	
Xu 2020	Adult	Allo	pts monitored serially for von Willebrand Factor levels	Pulmonary hypertension	23	39%	56	7%	32%
**Pulmonary Hemorrhage**									
Heybeli 2020	Adult	Allo	no restrictions	Diffuse alveolar hemorrhage	84	5%	1367	NR	
Jodele 2020	Peds	Both	all ECU	Pulmonary interstitial bleeding	64	11%	502	NR	
Jodele 2014b	Both	Allo	all ECU	Pulmonary interstitial bleeding	6	17%	NR	NR	
Schoettler 2019	Peds	Both	Mostly neuroblastoma (78%)	Severe pulmonary hemorrhage	9	11%	NR	NR	
**Pleurocentesis**									
Jodele 2020	Peds	Both	all ECU	Pleurocentesis	64	3%	502	NR	
Dandoy 2021	Peds	Both	no restrictions	Pleural effusion req. pleurocentesis or chest tube	98	4%	516	0.8%	3%
**Respiratory Failure (without detail on type of ventilation)**									
Jodele 2014a	Both	Allo	no restrictions	Respiratory failure	39	33%	51	6%	27%
Schoettler 2019	Peds	Auto	Mostly neuroblastoma (78%)	Mechanical ventilation	9	44%	NR	NR	
Jodele 2018	Peds	Auto	Neuroblastoma	Mechanical ventilation	13	15%	NR	NR	
**Non-Invasive Ventilation**									

Author Year	Adult / Peds	HSCT Type	Special Population	Complication	TMA (N)	TMA %	non-TMA (N)	non-TMA %	Diff
Imus 2020	Adult	Allo	all pts received high-dose post-HSCT cyclophosphamide for GVHD prophylaxis	Supplemental oxygen	9	11%	669	NR	
Dandoy 2021	Peds	Both	no restrictions	Respiratory failure requiring noninvasive ventilation	98	30%	516	6%	23%
**Invasive Mechanical Ventilation**									
Li 2019	Adult	Allo	no restrictions	Intubation/mechanical ventilation	192	29%	1953	NR	
Dandoy 2021	Peds	Both	no restrictions	Intubation/mechanical ventilation	98	26%	516	6%	19%
Jodele 2020	Peds	Both	all ECU	Intubation/mechanical ventilation	64	41%	502	NR	
Imus 2020	Adult	Allo	all pts received high-dose post-HSCT cyclophosphamide for GVHD prophylaxis	Intubation/mechanical ventilation	9	44%	669	NR	

Allo = allogeneic, Auto = autologous, ECU = eculizumab, DNA = deoxyribonucleic acid, GVHD = graft versus host disease, HSCT = hematopoietic stem cell transplant, NR = not reported, PE = plasma exchange, Peds = pediatric, pts = patients, SIR = sirolimus, TAC = tacrolimus, TMA = thrombotic microangiopathy

Table S4. Cardiovascular Symptoms

Author Year	Adult / Peds	HSCT Type	Special Population	Complication	TMA (N)	TMA %	non-TMA (N)	non-TMA %	Diff
**Poor Cardiac Function**									
Rosenthal 2011	Peds	Allo	all SIR & TAC	Poor cardiac function	10	20%	31	NR	
**Pericarditis**									
De Fontbrune 2015	Both	Allo	all ECU; severe HSCT-TMA (neuro and/or renal involvement)	Pericarditis	12	17%	NR	NR	
**Pericardial Effusion**									
Dandoy 2021	Peds	Both	no restrictions	Pericardial effusion req. pericardiocentesis	98	9%	516	0.6%	9%
Jodele 2014a	Both	Allo	no restrictions	Pericardial effusion	39	38%	51	20%	19%
Jodele 2014b	Both	Allo	all ECU	Pericardial effusion	6	67%	NR	NR	
Jodele 2018	Peds	Auto	Neuroblastoma	Pericardial effusion	13	92%	NR	NR	
Cox 2017	Peds	Both	no restrictions	Pericardial effusion	6	67%	113	19%	48%
Schoettler 2019	Peds	Auto	Mostly neuroblastoma (78%)	Pericardial effusion	9	56%	NR		
**Cardiac Tamponade**									
Jodele 2014b	Both	Allo	all ECU	Pericardiocentesis for cardiac tamponade	6	17%	NR	NR	
Jodele 2020	Peds	Both	all ECU	Pericardiocentesis for cardiac tamponade	64	19%	502	NR	
Schoettler 2019	Peds	Auto	Mostly neuroblastoma (78%)	Pericardiocentesis for cardiac tamponade	9	22%	NR	NR	

Allo = allogeneic, Auto = autologous, ECU = eculizumab, DNA = deoxyribonucleic acid, GVHD = graft versus host disease, HSCT = hematopoietic stem cell transplant, NR = not reported, PE = plasma exchange, Peds = pediatric, pts = patients, SIR = sirolimus, TAC = tacrolimus, TMA = thrombotic microangiopathy

Table S5. Neurological Symptoms

Author Year	Adult / Peds	HSCT Type	Special Population	Complication	TMA (N)	TMA %	non-TMA (N)	non-TMA %	Diff
**Encephalopathy**									
Jodele 2020	Peds	Both	all ECU	Posterior reversible encephalopathy syndrome	64	9%	502	NR	
Jodele 2020	Peds	Both	all ECU	Mental status changes due to encephalopathy	64	13%	502	NR	
Jodele 2014b	Both	Allo	all ECU	Posterior reversible encephalopathy syndrome	6	17%	NR	NR	
Imus 2020	Adult	Allo	all pts received high-dose post-HSCT cyclophosphamide for GVHD prophylaxis	Encephalopathy	9	33%	669	NR	
Jodele 2018	Peds	Auto	Neuroblastoma	Posterior reversible encephalopathy syndrome	13	15%	NR	NR	
**Seizures**									
Imus 2020	Adult	Allo	all pts received high-dose post-HSCT cyclophosphamide for GVHD prophylaxis	Seizures	9	11%	669	NR	
Jodele 2014b	Both	Allo	all ECU	Seizures	6	17%	NR	NR	
Jodele 2020	Peds	Both	all ECU	Seizures	64	19%	502	NR	
**Neurological Symptoms**									
Sarkodee-Adoo 2003	Adult	Allo	no restrictions	Neurological symptoms	11	0%	44	NR	
Shimoni 2004	Adult	Allo	no restrictions	Neurological symptoms thought to be predominantly due to TMA	22	9%	125	NR	
Jodele 2014a	Both	Allo	no restrictions	Neurological symptoms	39	23%	51	8%	15%
Li 2019	Adult	Allo	no restrictions	Neurological symptoms	192	29%	1953	NR	
Kraft 2019	Adult	Allo	no restrictions	Neurological symptoms	65	29%	595	NR	
Jodele 2020	Peds	Both	all ECU	Neurological symptoms	64	33%	502	NR	
de Fontbrune 2015	Both	Allo	all ECU; severe HSCT-TMA (neuro and/or renal involvement)	Neurological symptoms	12	33%	NR	NR	
Martinez 2005	Both	Allo	no restrictions	Neurological symptoms	68	40%	153	24%	16%
Rudoni 2018	Adult	Allo	all ECU	Neurological symptoms	10	40%	NR	NR	

Author Year	Adult / Peds	HSCT Type	Special Population	Complication	TMA (N)	TMA %	non-TMA (N)	non-TMA %	Diff
Oran 2007	Adult	Allo	all engrafted w/ non-T-cell depleted transplant and had TAC-based GVHD prophylaxis	Neurological symptoms (mental status or headache)	66	42%	1153	NR	
Shimoni 2004	Adult	Allo	no restrictions	Neurological symptoms	22	50%	125	NR	
Imus 2020	Adult	Allo	all pts received high-dose post-HSCT cyclophosphamide for GVHD prophylaxis	Neurological symptoms	9	56%	669	NR	
Nakamae 2006	Adult	Allo	no restrictions	Neurological symptoms	22	64%	101	NR	
Hahn 2004	Both	Allo	no restrictions	Neurological symptoms	19	74%	209	NR	
Schoettler 2019	Peds	Auto	Mostly neuroblastoma (78%)	Neurological symptoms	9	11%	NR	NR	
**Bleeding and Stroke**									
Jodele 2020	Peds	Both	all ECU	Subdural hematoma req. draining	64	2%	502	NR	
Jodele 2020	Peds	Both	all ECU	CNS bleeding	64	6%	502	NR	
Imus 2020	Adult	Allo	all pts received high-dose post-HSCT cyclophosphamide for GVHD prophylaxis	Stroke	9	11%	669	NR	
Jodele 2018	Peds	Auto	Neuroblastoma	Intracranial bleeding	13	15%	NR	NR	

Allo = allogeneic, Auto = autologous, ECU = eculizumab, DNA = deoxyribonucleic acid, GVHD = graft versus host disease, HSCT = hematopoietic stem cell transplant, NR = not reported, PE = plasma exchange, Peds = pediatric, pts = patients, SIR = sirolimus, TAC = tacrolimus, TMA = thrombotic microangiopathy

Table S6. Gastrointestinal and Hepatic Symptoms

Author Year	Adult / Peds	HSCT Type	Special Population	Complication	TMA (N)	TMA %	non-TMA (N)	non-TMA %	Diff
**Gastrointestinal Symptoms**									
El-Bietar 2015	Peds	Allo	intestinal GVHD	Nausea/vomiting	15	67%	21	76%	-10%
El-Bietar 2015	Peds	Allo	intestinal GVHD	Diarrhea	15	93%	21	90%	3%
**Abdominal Pain**									
El-Bietar 2015	Peds	Allo	intestinal GVHD	Abdominal pain	15	93%	21	76%	17%
**Gastrointestinal Bleeding**									
Heybeli 2020	Adult	Allo	no restrictions	Gastrointestinal bleeding	84	8%	1367	NR	
Jodele 2014a	Both	Allo	no restrictions	Significant GI bleed	39	26%	51	4%	22%
Jodele 2020	Peds	Both	all ECU	Lower GI bleeding	64	36%	502	NR	
Xu 2020	Adult	Allo	pts monitored serially for von Willebrand Factor levels	Severe GI bleed	23	74%	56	32%	42%
El-Bietar 2015	Peds	Allo	intestinal GVHD	Transfusion for GI bleeding	15	60%	21	10%	50%
**Liver Injury**									
Rosenthal 2011	Peds	Allo	all SIR & TAC	Poor hepatic function	10	10%	31	NR	
Rudoni 2018	Adult	Allo	all ECU	Liver injury	10	30%	NR	NR	
Xu 2020	Adult	Allo	pts monitored serially for von Willebrand Factor levels	Liver damage	23	78%	56	48%	30%

Allo = allogeneic, Auto = autologous, ECU = eculizumab, DNA = deoxyribonucleic acid, GVHD = graft versus host disease, HSCT = hematopoietic stem cell transplant, NR = not reported, PE = plasma exchange, Peds = pediatric, pts = patients, SIR = sirolimus, TAC = tacrolimus, TMA = thrombotic microangiopathy

Table S7. Other Symptoms

Author Year	Adult / Peds	HSCT Type	Special Population	Complication	TMA (N)	TMA %	non-TMA (N)	non-TMA %	Diff
**Multi-organ Dysfunction**									
Rudoni 2018	Adult	Allo	all ECU	Multiorgan dysfunction	10	70%	NR	NR	
Jodele 2020	Peds	Both	all ECU	Multiorgan dysfunction syndrome	64	84%	502	NR	
**Retinitis**									
de Fontbrune 2015	Both	Allo	all ECU; severe HSCT-TMA (neuro and/or renal involvement)	Organ injury: Retinitis	12	8%	NR	NR	

Allo = allogeneic, Auto = autologous, ECU = eculizumab, DNA = deoxyribonucleic acid, GVHD = graft versus host disease, HSCT = hematopoietic stem cell transplant, NR = not reported, PE = plasma exchange, Peds = pediatric, pts = patients, SIR = sirolimus, TAC = tacrolimus, TMA = thrombotic microangiopathy

Table S8. Infections

Author Year	Adult / Peds	HSCT Type	Special Population	Complication	TMA (N)	TMA rate	non-TMA (N)	non-TMA rate	Diff
**Overall Infections**									
Dandoy 2021	Peds	Both	no restrictions	2 or more bloodstream infections in 100d	98	17%	418	5%	13%
Dandoy 2021	Peds	Both	no restrictions	One or more bloodstream infections in 100d	98	39%	418	21%	18%
De Fontbrune 2015	Both	Allo	all ECU; severe HSCT-TMA (neuro and/or renal involvement)	Infection being treated at TMA diagnosis	12	50%	NR	NR	
El-Bietar 2015	Peds	Allo	intestinal GVHD	Co-infections	15	53%	21	48%	6%
Oran 2007	Adult	Allo	all engrafted w/ non-T-cell depleted transplant and had TAC-based GVHD prophylaxis	Active infection	66	70%	1153	NR	
Gavriilaki 2020	Peds	Allo	Case-control study: TMA (no restrictions), control group consists of HSCT donors with DNA samples and HSCT pts w/o TMA	Infection	40	78%	40	73%	5%
Rudoni 2018	Adult	Allo	all ECU	Active infection	10	80%		NR	
Xu 2020	Adult	Allo	pts monitored serially for von Willebrand Factor levels	Severe infection	23	83%	56	34%	49%
**Specific Infections**									
Imus 2020	Adult	Allo	all pts received high-dose post-SCT cyclophosphamide for GVHD prophylaxis	Cytomegalovirus infection (CMV)	9	11%	669	NR	
Kraft 2019	Adult	Allo	no restrictions	CMV reactivation / end-organ disease	65	54%	595	26%	28%
Imus 2020	Adult	Allo	all pts received high-dose post-HSCT cyclophosphamide for GVHD prophylaxis	Clostridium difficile infection	9	11%	669	NR	
Imus 2020	Adult	Allo	all pts received high-dose post-HSCT cyclophosphamide for GVHD prophylaxis	Enterococcus infection	9	22%	669	NR	
Imus 2020	Adult	Allo	all pts received high-dose post-HSCT cyclophosphamide for GVHD prophylaxis	Staphylococcus infection	9	22%	669	NR	
Jodele 2020	Peds	Both	all ECU	Meningitis	64	2%	502	NR	
**Fever**									
Xu 2020	Adult	Allo	pts monitored serially for von Willebrand Factor levels	Fever	23	70%	56	23%	46%

Allo = allogeneic, Auto = autologous, ECU = eculizumab, DNA = deoxyribonucleic acid, GVHD = graft versus host disease, HSCT = hematopoietic stem cell transplant, NR = not reported, PE = plasma exchange, Peds = pediatric, pts = patients, SIR = sirolimus, TAC = tacrolimus, TMA = thrombotic microangiopathy

Table S9. GVHD

Author Year	Adult / Peds	HSCT Type	Special Population	Complication	TMA (N)	TMA %	non-TMA (N)	non-TMA %	Diff
**Acute GVHD after TMA**									
Wall 2018	Adult	Allo	HSCT with GVHD	Acute GVHD post- TMA	84	10%	40		
Jodele 2020	Peds	Both	all ECU	Acute GVHD post-TMA	64	23%	502	NR	
**Acute GVHD concurrent with TMA (any grade)**									
Dandoy 2021	Peds	Both	no restrictions	Acute GVHD (any grade)	98	32%	516	26%	6%
de Fontbrune 2015	Both	Allo	all ECU; severe HSCT-TMA (neuro and/or renal involvement)	Acute GVHD (any grade)	12	33%	NR	NR	
Imus 2020	Adult	Allo	all pts received high-dose post-HSCT cyclophosphamide for GVHD prophylaxis	Acute GVHD (any grade)	9	33%	669	NR	
Heybeli 2020	Adult	Allo	no restrictions	Acute GVHD (any grade)	84	46%	1367	NR	
Jodele 2020	Peds	Both	all ECU	Acute GVHD (any grade)	64	47%	502	NR	
Jodele 2014b	Both	Allo	all ECU	Acute GVHD (any grade)	6	50%		NR	
Rosenthal 2011	Peds	Allo	all SIR & TAC	Acute GVHD (any grade)	10	50%	31	NR	
Xu 2020	Adult	Allo	pts monitored serially for von Willebrand Factor levels	Acute GVHD (any grade)	23	57%	56	46%	10%
Rudoni 2018	Adult	Allo	all ECU	Acute GVHD (any grade)	10	70%	NR	NR	
**Acute GVHD concurrent with TMA (grades 2-4)**									
Postalcioglu 2018	Adult	Allo	no restrictions	Acute GVHD (grade 2-4)	766	35%	1224	25%	10%
Erbey 2010	Peds	Allo	no restrictions	Acute GVHD (grade 2-4)	3	67%	47	NR	
Kraft 2019	Adult	Allo	no restrictions	Acute GVHD (grade 2-4)	65	71%	595	50%	21%
Gavriilaki 2020	Peds	Allo	Case-control study: TMA (no restrictions), control group consists of HSCT donors with DNA samples and HSCT pts w without TMA	Acute GVHD (grade 2-4)	40	83%	40	90%	-8%
Sarkodee-Adoo 2003	Adult	Allo	no restrictions	Acute GVHD (grade 2-4)	11	100%	44	NR	
**Acute GVHD concurrent with TMA (grades 3-4)**									
Sartain 2019	Peds	Allo	all PE treated	Acute GVHD (grade 3-4)	15	40%	NR	NR	
Hahn 2004	Both	Allo	no restrictions	Acute GVHD (grade 3-4)	19	47%	209	25%	22%

Author Year	Adult / Peds	HSCT Type	Special Population	Complication	TMA (N)	TMA %	non-TMA (N)	non-TMA %	Diff
Martinez 2005	Both	Allo	no restrictions	Acute GVHD (grade 3-4)	68	41%	153	16%	25%
**Bleeding Related to GVHD**									
Heybeli 2020	Adult	Allo	no restrictions	Severe bleeding related to gastrointestinal GVHD	84	8%	1367	NR	
**Chronic GVHD**									
Rosenthal 2011	Peds	Allo	all SIR & TAC	Chronic GVHD	10	50%	31	NR	
Gavriilaki 2020	Peds	Allo	Case-control study: TMA (no restrictions), control group consists of HSCT donors with DNA samples and HSCT pts without TMA	Chronic GVHD (grade 2-4)	40	53%	40	60%	-8%
Heybeli 2020	Adult	Allo	no restrictions	Chronic GVHD	84	88%	1367	NR	

Allo = allogeneic, Auto = autologous, ECU = eculizumab, DNA = deoxyribonucleic acid, GVHD = graft versus host disease, HSCT = hematopoietic stem cell transplant, NR = not reported, PE = plasma exchange, Peds = pediatric, pts = patients, SIR = sirolimus, TAC = tacrolimus, TMA = thrombotic microangiopathy
